# Evolution of Predator Dispersal in Relation to Spatio-Temporal Prey Dynamics: How Not to Get Stuck in the Wrong Place!

**DOI:** 10.1371/journal.pone.0054453

**Published:** 2013-02-11

**Authors:** Justin M. J. Travis, Stephen C. F. Palmer, Steven Coyne, Alexandre Millon, Xavier Lambin

**Affiliations:** 1 Institute of Biological and Environmental Sciences, University of Aberdeen, Aberdeen, United Kingdom; 2 Institut Méditerranéen d’Ecologie et de Paléoécologie, Centre National de la Recherche Scientifique, Aix-en-Provence, France; McGill University, Canada

## Abstract

The eco-evolutionary dynamics of dispersal are recognised as key in determining the responses of populations to environmental changes. Here, by developing a novel modelling approach, we show that predators are likely to have evolved to emigrate more often and become more selective over their destination patch when their prey species exhibit spatio-temporally complex dynamics. We additionally demonstrate that the cost of dispersal can vary substantially across space and time. Perhaps as a consequence of current environmental change, many key prey species are currently exhibiting major shifts in their spatio-temporal dynamics. By exploring similar shifts *in silico*, we predict that predator populations will be most vulnerable when prey dynamics shift from stable to complex. The more sophisticated dispersal rules, and greater variance therein, that evolve under complex dynamics will enable persistence across a broader range of prey dynamics than the rules which evolve under relatively stable prey conditions.

## Introduction

Dispersal is fundamental to a species’ ability to exploit its environment, influencing spatial population dynamics and gene flow [1]. Dispersal behaviour is however not a fixed trait, and there is now compelling empirical evidence that dispersal is expressed in a context-dependent manner and that it can evolve on an ecological timescale [2]–[4]. Thus both dispersal plasticity over short time-scales and dispersal evolution over longer time-scales may contribute to the persistence of populations in circumstances initially inimical to persistence. Improving understanding of eco-evolutionary dispersal dynamics through modelling is crucial for predicting and managing biodiversity responses to rapid environmental change [5].

The majority of theory on the evolution of dispersal has focussed on understanding how individuals of a single species behave in response to their environment and the density of conspecifics [6]–[10] Whilst earlier theoretical work focussed almost exclusively on modelling the evolution of emigration propensities, in recent years there has been a rapid move towards including greater detail and, in particular, increased attention has been given to modelling the transfer and settlement phases of dispersal [11]. A number of interesting studies have explored the evolution of dispersal within the context of trophic interactions [12]–[16]: typically these models have assumed either global dispersal (where individuals can move to any habitat patch on the landscape) or local dispersal (where individuals are constrained to dispersing to a neighbouring habitat patch) and they have mostly focussed on understanding the evolution of emigration rates of either prey or predators (or both) in systems where the dynamics of prey and predator are interdependent. Despite these notable exceptions, there remains relatively little work exploring dispersal evolution in the context of inter-specific interactions and certainly there is a lack of work that has adopted the more mechanistic approach to dispersal evolution modelling that has developed recently in single-species theory.

In trophic interactions, dispersal allows prey to escape regions where predation risk is high [16] and similarly allows consumers to escape a place where prey density is not sufficient for breeding and/or surviving, and thus track a resource that is fluctuating both temporally and spatially [17]. Considerable existing theory on the evolution of dispersal has already demonstrated the key role that temporal variability in patch quality plays in selecting for higher rates of dispersal [7], [18], and that this effect is stronger under temporally uncorrelated variability in local conditions (white noise) than when there is temporal autocorrelation (red noise) [19]. Theory has similarly emphasised the importance of the extent and pattern of spatial variability in the environment in dispersal evolution, including in the maintenance of dispersal polymorphisms [20]–[23]. Typically we should expect higher rates of dispersal to evolve when there is high spatial autocorrelation (i.e. good conditions tend to occur close to one another) than when good and poor conditions occur randomly through space [19], [24]. However, no investigation has simultaneously included spatial and temporal autocorrelation or explored how reaction norms of a predator’s dispersal should evolve according to prey dynamics, potentially enabling mobile predators to adaptively utilise spatio-temporal information on prey densities. Yet, given spatio-temporal variability in resource availability, emigration rates and stopping rules responsive to variation in prey density should not only outperform fixed dispersal strategies but also better reflect biological reality. Classical ecological theory on spatially extended trophic interactions, while also well developed, concentrates on the role of rates of dispersal in facilitating the persistence of dynamically coupled predators and prey and how this may generate complex spatio-temporal dynamics [25]–[29]. However, neither evolutionary nor ecological theories explore how evolution might shape the dispersal strategies of the many predators that exploit prey populations with different types of spatio-temporal dynamics, but that are sufficiently scarce such that their intake does not appreciably affect prey dynamics (but see [30] for the special case of perfectly regular cycles).

Furthermore, theory offers no guidance on how changes in spatio-temporal prey dynamics might affect the populations of predators confronted with such novel circumstances. Yet, the distribution of resources exploited by many natural enemies (predators/parasites/herbivores) is changing dramatically, perhaps owing to environmental change. One striking example is the broad-scale and substantial dampening in the spatio-temporal dynamics of herbivorous moths, gamebirds, lagomorphs and small rodents with cyclic dynamics reported from across Europe [31]–[33]. Predicting whether guilds of predators will be able to respond to these changes requires a new understanding of how a predator’s dispersal rules (including emigration rate and context-dependent patch selection) evolve in response to different spatio-temporal prey dynamics, and to assess how predators which have evolved a dispersal strategy in one spatio-temporal prey environment will fare when their prey’s spatio-temporal dynamics change.

Here we will use a spatially-extended, time-delayed discrete-time Ricker equation to explore the impact of spatio-temporal variability in prey resources on dispersal evolution and resulting population dynamics in a predator species. This model can generate stable and travelling waves or more complex dynamics including spatial chaos, all of which have been described in real prey species [34]. We will extend a well-understood, individual-based model to simulate a predator’s dynamics and, drawing on recent developments in modelling dispersal and its evolution in single-species models [11], [35], [36], will explore how emigration rules and context-dependent settlement decisions evolve in response to the underlying prey dynamics.

## The Model

Here, we combine approaches previously used to explore potentially complex spatial population dynamics of a species with those developed to investigate the evolution of dispersal. We model the dynamics of a prey population using a deterministic lattice model and link this to an individual-based model of predators which exploit the prey. We assume that the predator is involved in “bottom-up” resource-consumer dynamics, whereby the predator population dynamics is driven by the dynamics of its main prey with no feedback from predator to prey. Each predator carries ‘genes’ that determines its dispersal rules and thus, we are able to explore how predator dispersal evolves in response to its prey’s spatial population dynamics.

### Prey Population Dynamics

#### Within patch prey dynamics

In each cell, we simulate population dynamics using the time-delayed discrete-time Ricker equation [37], [38]:
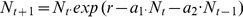
(1)


Where *r* is the reproductive rate, and *a_1_* and *a_2_* determine the strength of direct and delayed density dependence, respectively.

#### Prey dispersal

Following the within-patch dynamics, we simulate prey dispersal. A proportion of individuals (*m*) emigrates from the natal cell, such that *m*/8 moves to each of the eight neighbouring cells.

By varying *r*, *a_1_*, *a_2_* and *m*, a wide range of spatio-temporal dynamics can be generated (see Fig. S1), some of which bears qualitative resemblance to the dynamics of real prey populations. Here, we follow [38] in using default values of *a_1_* = 0.05, *a_2_* = 0.05 and *m* = 0.1. As an example, when *r* = 2.2, an isolated population, once transients die out, fluctuates with regular cycles which are repeated every four years, roughly analogous to microtine rodent dynamics [37]. The scale of spatial synchrony covaries with underlying temporal dynamics. When such a model is spatially extended, travelling waves emerge, which again resemble those observed in many oscillating populations of voles, grouse and moths [38]–[40].

### Predator Population and Evolutionary Dynamics

We model the predator population using an individual-based approach which is an extension on those described elsewhere [41].

#### Within-patch predator dynamics

Subpopulation dynamics in each cell are described by a formulation based on [42]. Each individual in the subpopulation at time *t* gives birth to a number of offspring drawn at random from a Poisson distribution with mean *μ* defined as:
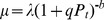
(2)


Here, *λ* specifies the intrinsic rate of increase, *q* relates to patch quality, *P_t_* is the number of predators present in the patch at time *t* and *b* governs the type of competition. Here, we use *b* = 1, such that the competition is ‘contest’. The parameter *q* is calculated from the following expression:
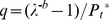
(3)where *P_t_* ^*^ is the predator subpopulation carrying capacity, which depends on the local abundance of prey *N_t_*. For simplicity we assume that *P_t_* ^*^ = *N_t_* in each grid cell. Drawing the number of offspring born to each adult from a Poisson distribution introduces a degree of demographic stochasticity.

#### Predator dispersal

Dispersal of predators follows dispersal of prey; hence dispersing predators settle in a cell whose prey density reflects the resource available to them when they subsequently breed. Asexually reproducing, haploid individuals carry ‘genes’ that specify their dispersal strategy. Most previous theory exploring dispersal evolution has focused on a density-independent emigration rate and, to root our results in this large body of theory, we start by doing the same. In this simplest case, each individual carries a single ‘gene’ that specifies the probability that the individual emigrates. We assume unlimited genetic variation such that this dispersal gene can take any value between 0.0 and 1.0. A cost of dispersal is applied to all emigrants such that they die with increased probability *c* relative to non-dispersers. Dispersing individuals move with equal likelihood to any one of the nearest eight neighbouring cells and stop there. Offspring inherit their emigration probability gene from their parent but with a probability *z* of mutation (here fixed at 0.01). When a mutation occurs, it is equally likely to increase or decrease dispersal probability. Its magnitude is drawn from a normal distribution with zero mean and variance = 0.04, but the mutation is applied to the logit-transformed gene value so that the resulting genetic dispersal probability is constrained to be between 0 and 1.0.

Extending upon this standard framework to explore more complex and realistic behaviours including condition-dependent settling rules, we adapt the model such that predators can evolve a flexible strategy whereby they can step through multiple cells during dispersal (no constraints on direction were applied), assessing each one and deciding whether to settle in it. Initially, we make the probability of stopping in a patch dependent only upon current prey density in that patch. This requires an individual to carry two additional genes - the intercept and slope of the relationship of logit(*P_stop_*) vs. prey density. The two new stopping (or arrival) genes mutate together as a pair (i.e. neither or both were subject to mutation), but independently of the emigration (or departure) gene. Subsequently, we further develop the stopping probability to be a function of the prey-to-predator ratio in the current square. Dispersing juveniles are processed in a random order, as early arrivals at a square are more likely to settle there than later arrivals. If a stopping rule gene is selected for mutation, the new value is drawn from a normal distribution centred on the current value and of specified variance (fixed here at 100 for the intercept and 25 for the slope). The mortality cost of dispersal is now applied on a per-step basis, *c_step_*, which remains constant throughout the dispersal process.

### Simulation Experiments

All our experiments were conducted on a lattice of dimension 50×50, which had reflecting boundaries, and genes were initialised to random values. There were two parts to our simulation experiments. First, we ran a large set of simulations to establish how predator dispersal evolves across a broad range of prey dynamics. These simulations were run both for the case where only emigration rate is allowed to evolve and for the extended model where emigration rate evolves jointly with the stopping rule. We systematically varied reproductive rate of prey *r* from 0.5 to 4.0 in increments of 0.25 and *c* from 0.01 to 0.05 in increments of 0.01, plus 0.10 and 0.20, and, for each combination, ran 5 independent simulations. We allowed the model to run for 1000 generations and then collected data on the population mean and inter-individual variation in predator dispersal strategies. In test simulations, we found that a quasi-equilibrium predator dispersal strategy had generally been reached by 500 generations, so running for 1000 time steps to pass the transient stage was conservative.

Second, in order to illuminate the impact of changes in prey dynamics such as those reported is a suite of recent studies [31]–[33] on predators with dispersal strategies evolved in different environments, we conducted experiments to explore how well (or poorly) a predator fares when there is an abrupt change in the spatio-temporal prey dynamics. We modelled three different conditions for generating respectively stable (*r* = 1.5), cyclic (*r* = 2.5) and chaotic (*r* = 3.5) prey dynamics. As in the previous set of simulations, predators were allowed 1000 generations to reach quasi-equilibrium. We then translocated some predator individuals from the prey landscape on which they had evolved to one that was empty of predators, but where the prey dynamics were different. We conducted these transplantations in two ways: first, we introduced 1000 individuals all of which were given the mean dispersal rules that had evolved in the source predator population; second, we sampled 1000 individuals at random from the predator population and introduced those – thus, we retained some genetically-based inter-individual variability. In both cases, translocated individuals were introduced to locations selected at random. Also, in both cases, once we had made the transplantation, we did not allow mutations to affect dispersal rules. So, in the first case, there was no evolution. In the second case, there could be selection on the initial variation, but there were no new mutations. We allowed the predator population 20 generations to adapt to the new conditions and repopulate the landscape and then collected data on both the predator population abundance and on the mean dispersal rules of the predators during the next 10 generations.

## Results

### (a) Evolved Predator Dispersal Strategy According to Spatio-temporal Dynamics of the Prey

**Figure 1:**
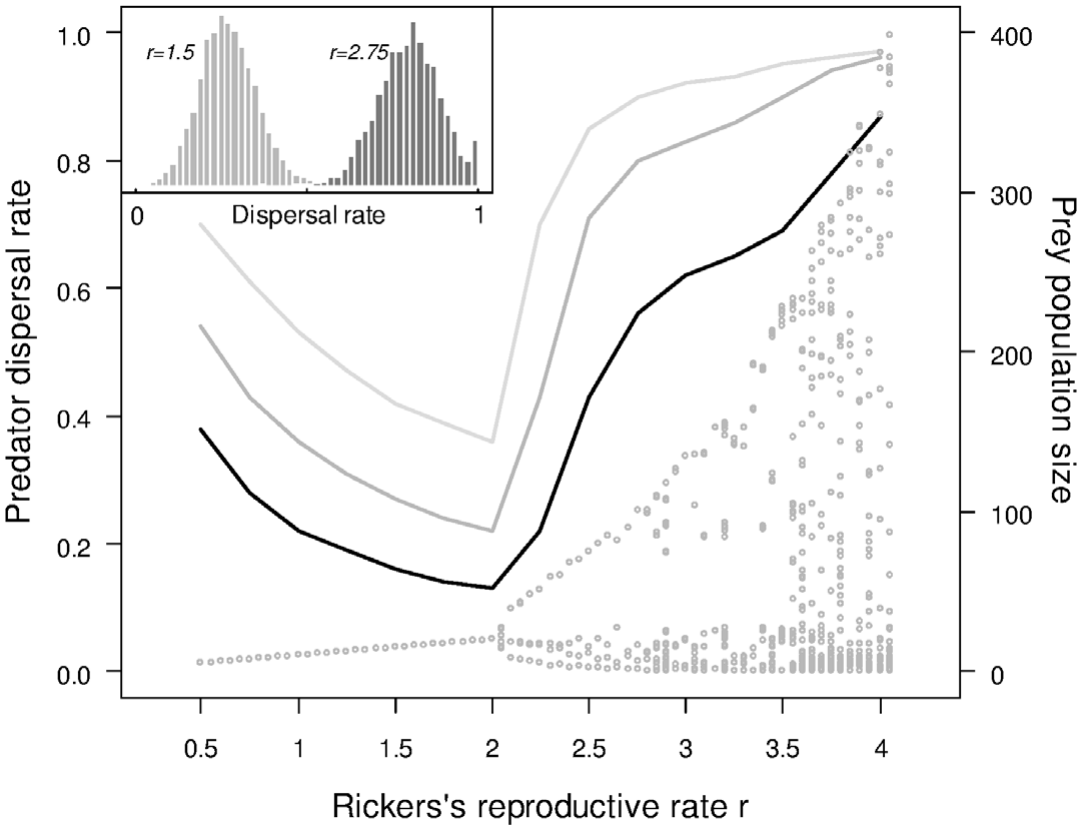
Evolution of predator emigration probability. Average predator emigration probabilities obtained after the model stabilized (left y-axis) for a range of *r* between 0.5 and 4.0 (by increments of 0.5). The three lines represent three different mortality costs of dispersal imposed on predators (from light grey to black: *c* = 0.05, 0.1, 0.2 respectively, see Methods). The bifurcation diagram shows the distribution of stable limits of the prey dynamics for the range of *r* values (right y-axis).The extent of variation in predator emigration probability at the population level is shown for two sets of simulations in the inserted panel (cost of dispersal *c* = 0.1 for both).

The spatio-temporal dynamics of the prey population varied according to the prey’s reproductive rate (*r*) (Fig. S1). For *r* <2.0, the dynamics were stable in space and time, and as *r* increased above 2.0 they became increasingly complex progressing through a cyclic regime where there were clear spatial travelling wave structures to a regime where the prey density within a patch exhibited much more complex fluctuations and the spatial structure of high prey density was more chaotic in nature. These prey dynamics exerted a strong influence on the evolution of a predator’s dispersal (Fig. 1). There were two main features of the relationship between prey *r* and the predator’s evolved dispersal. First, as *r* increased from 0.5 up to 2.0, lower emigration probabilities evolved. Note that for this range of *r* values, there was an increase in the (still stable) equilibrium prey population size (see the bifurcation map in Fig. 1). In this region of parameter space, whatever the dispersal cost, we find qualitatively similar declines in emigration probability; dispersal is roughly half as frequent when *r* = 2.0 as it is when *r* = 0.5. As expected, dispersal probability always substantially reduced as the cost of dispersing increased.

Second, as the underlying prey dynamics switched from stable equilibrium to cyclic at the threshold *r* = 2.0, there was a rapid increase in the evolved emigration probability (Fig. 1). Regardless of the cost of dispersal, this rise was steepest as *r* increased from 2.0 to about 2.6. For the two lower costs of dispersal, as *r* increased beyond around 2.6, the rate of increase in evolved emigration probability decreased to approach an asymptote close to 1.0 (i.e. almost all individuals disperse).

**Figure 2:**
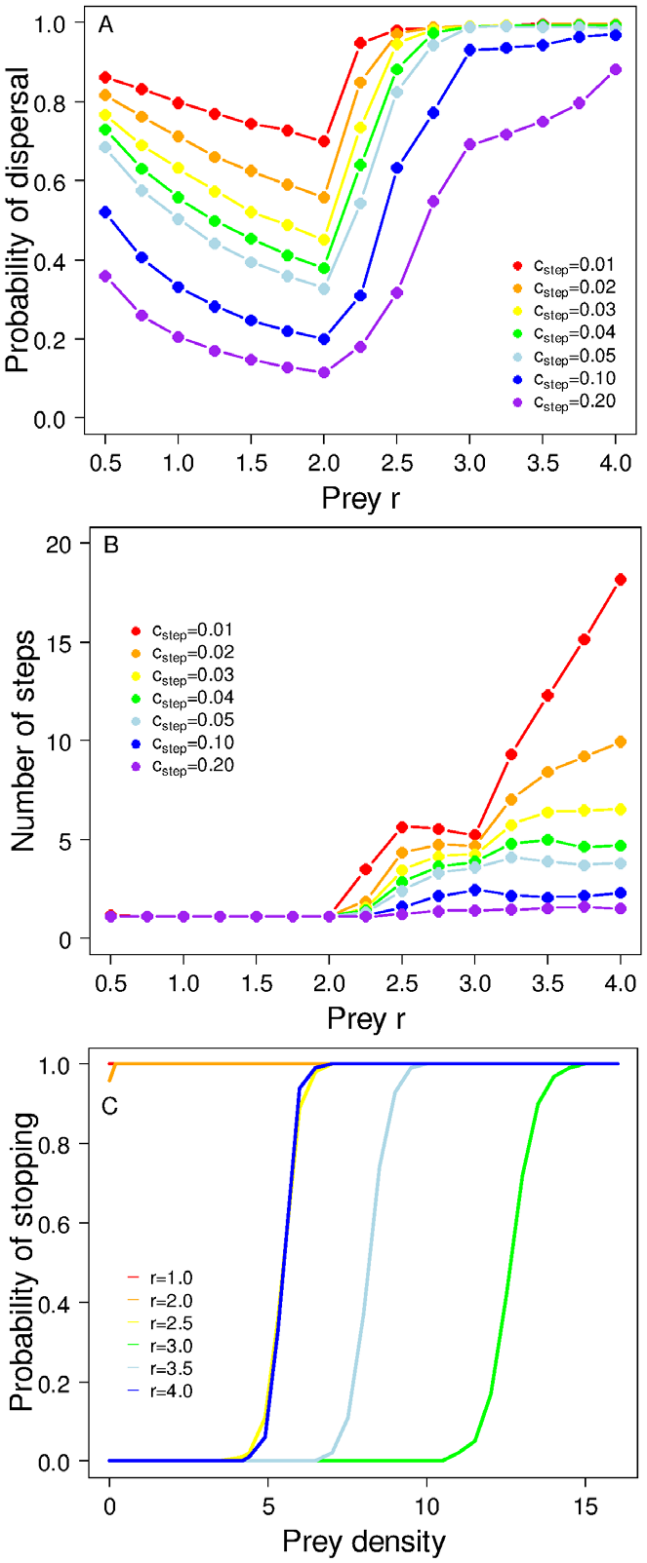
Evolution of a more complex predator dispersal strategy. A qualitatively similar response of dispersal to varying prey *r* is observed when predators can take multiple steps and evolve a stopping rule dependent on prey density. (A) Dispersal probability at seven levels of predator per-step dispersal mortality *c_step_*. (B) The mean number of steps taken by predators across the same range of *r* and *c_step_* as in (A). The number of steps taken by predators is a function of the rules that they have evolved, together with the spatio-temporal characteristics of the environment. (C) The mean stopping rules that evolve for a range of prey *r* at a moderate per-step mortality (*c_step_ = *0.02). The ‘slope’ gene determines the ‘steepness’ of the stopping threshold (the rate of change of probability with increasing prey density), and the ‘slope’ and ‘intercept’ genes together control the position of the stopping threshold in relation to prey density.

Where reaction norms that determine immigration rules were allowed to evolve jointly with emigration rate, we observed qualitatively similar responses of evolved emigration probability to the prey dynamics; this was true regardless of whether the predators stopped as a function of prey density alone (Fig. 2A) or as a function of the ratio between prey and predator density (data not shown). Interestingly, in simulations with a high per-step mortality cost of dispersal, we observed a sustained increase in emigration probability between *r* = 2.5 and *r* = 4.0. This is in contrast to results for lower costs where almost all individuals emigrated under more complex prey dynamics.

The mean number of steps taken by predators strongly depended upon the prey reproductive rate *r* and resulted from a combination of the evolved stopping rule and the spatial structure of the prey landscape. When predators responded only to prey density, in the stable prey region (*r* <2.0), they typically stopped at the first step (Fig. 2B–C; note red and orange lines in Fig. 2C show that stopping probability at *r* ≤2.0 was ∼1 and independent of prey density). In the cycling prey region (Fig. 2C; yellow/green lines), the stopping rule became discriminatory; predators tended to move for several steps until they found a cell where prey density was relatively high, and the threshold varied with prey behaviour, being much higher at *r* = 3.0 (green) than at *r* = 2.5 (yellow). As prey moved into the chaotic region (Fig. 2C; blue lines), the evolved threshold prey density started to fall again as the certainty with which high prey cells were likely to be encountered within a limited number of steps decreased.

**Figure 3:**
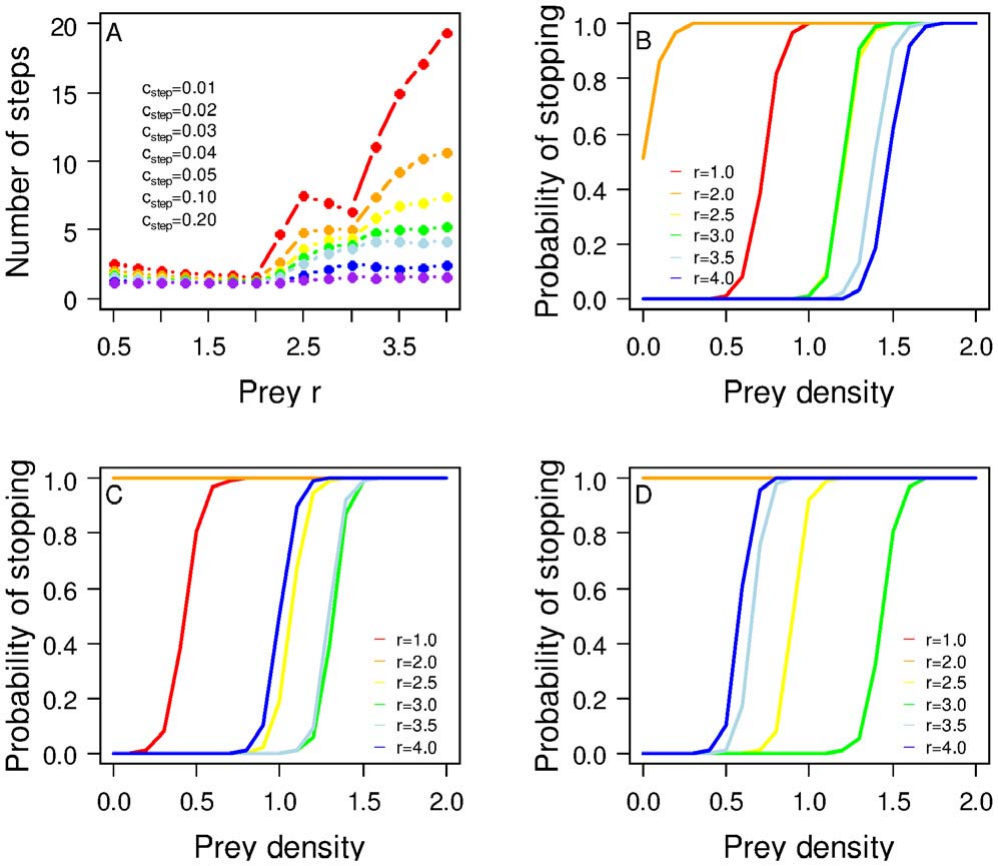
Predators are choosier and take more steps when the per-step cost of dispersal is low. Here we illustrate results obtained when the predators evolve a stopping rule that is sensitive to the ratio of prey per predator. The mean number of steps taken by predators is shown for a range of prey *r* and per-step mortality *c_step_* in A. In B, C and D, we show the mean stopping rules that evolve for *c_step_* = 0.02, 0.04 and 0.10, respectively.

Allowing the predators to evolve a stopping rule that accounts for the ratio between prey and predator density resulted in a slight increase in the mean number of dispersal steps (Fig. 3A). However, the evolved stopping rule changed considerably (compare Fig. 3B with 2C). At low per-step mortality, under stable and relatively low prey conditions (Fig. 3B; red line), the predators could afford to disperse until finding at least 0.5 prey units per predator present, but as prey density increased (orange line) even this low risk of mortality was not selected for, and most predators stopped immediately, whereas in the cyclic and chaotic prey regions, the stopping rules were very similar, and predators continued to move until 1.0 to 1.5 prey units per predator were encountered. However, as predator mortality was increased, the stopping rules changed to reflect the greater risk of taking more steps (Fig. 3C–D). Under stable prey conditions, predators tended to stop after one step, regardless of the prey density or how many competitors were present. In the cyclic prey region, the threshold prey per predator became strongly dependent on prey *r* (yellow and green lines), whereas it was independent of *r* at low mortality risk (coincident yellow and green lines in Fig 3B). In the chaotic prey region, the threshold levels of prey per predator declined sharply with increasing mortality risk, and predators tended to stop when only a moderate level of ∼0.5 prey units per predator was encountered.

In most previous models of dispersal evolution, a fixed cost of dispersing has typically been applied. For example, often a proportion of emigrants are assumed to suffer mortality before arriving at a destination patch. In our multi-step model, where individuals evolve a reaction norm to determine when they stop, we found that a strong spatial structure emerged in the realised cost of dispersing (Fig S2). An emigrant’s probability of mortality before selecting a destination patch was highest when it was born into a high density predator landscape where the prey population was collapsing. Conversely, mortality of dispersers was very low when they were born into areas with few predators and where prey were increasing in density.

**Figure 4:**
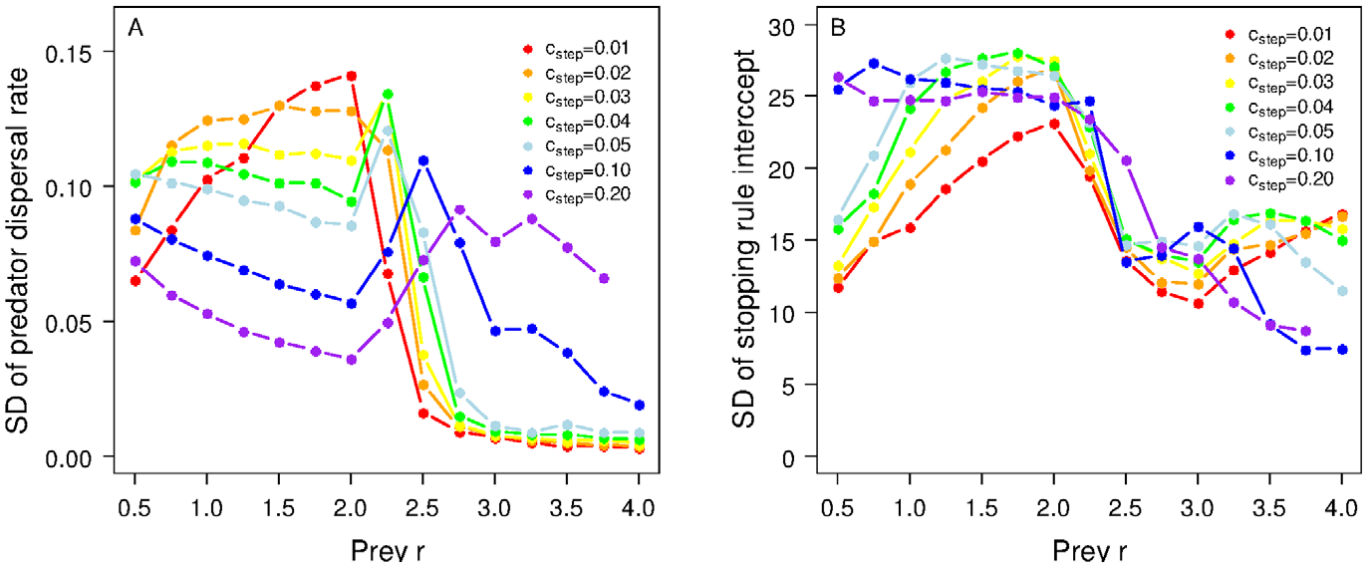
Within-population variability in dispersal rules depends upon spatio-temporal prey dynamics. As well as determining the mean dispersal rules, the underlying prey dynamics influence the emergent heterogeneity in dispersal rules. (A) The standard deviation in the emigration probability across a range of prey *r*. (B) The standard deviation in the intercept of the reaction norm that determines the stopping probability as a function of prey per predator. All parameters values are as for Fig 3. Note that where there are missing values it is due to the predator populations always going to extinction; this occurs when prey *r* and *c_step_* are both high.

Under all prey conditions and in both the experiments where only emigration probability evolved and those where emigration propensity evolved jointly with the stopping rule, we found some within-population diversity in predator dispersal strategies (see insert in Fig 1 and SDs for dispersal rules across a broad range of parameter space in Fig 4). Importantly, we found that, in addition to influencing the mean dispersal rules, the underlying spatio-temporal prey dynamics exerted a strong influence on the level of heterogeneity within the population. Typically, we found greatest within-population variability in both emigration probability and in the reaction norm for the stopping rules when prey *r* was in the cyclic region and lowest when the dynamics were more chaotic (higher prey *r*). This inter-individual variability becomes important within the context of changing prey dynamics, the results of which are described next.

### (b) Predator Response to Changed Prey Dynamics Transplant Experiments

**Figure 5:**
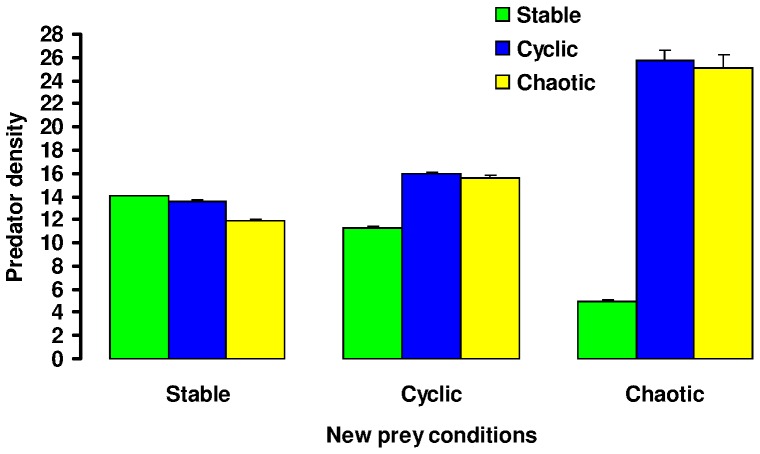
Typical results from a transplant experiment: the density of populations (predators per landscape cell) applying rules evolved under stable (prey *r* = 1.5), cyclic (*r* = 2.5) and complex (*r* = 3.5) prey dynamics when they are placed into each of those three conditions. It is clear that while the strategies that evolve under cyclic or complex prey dynamics prove quite robust within other prey environments, the strategy that evolves under stable prey dynamics results in substantially reduced population density when it is placed in a more complex prey landscape. Mean population sizes are calculated from the 20^th^ to 30^th^ generations following transplantation. Transplanted predators had genes equal to the population mean values, the stopping rule based on prey per predator was employed, and per-step mortality *c_step_* was fixed at 0.02 in all cases.

**Figure 6:**
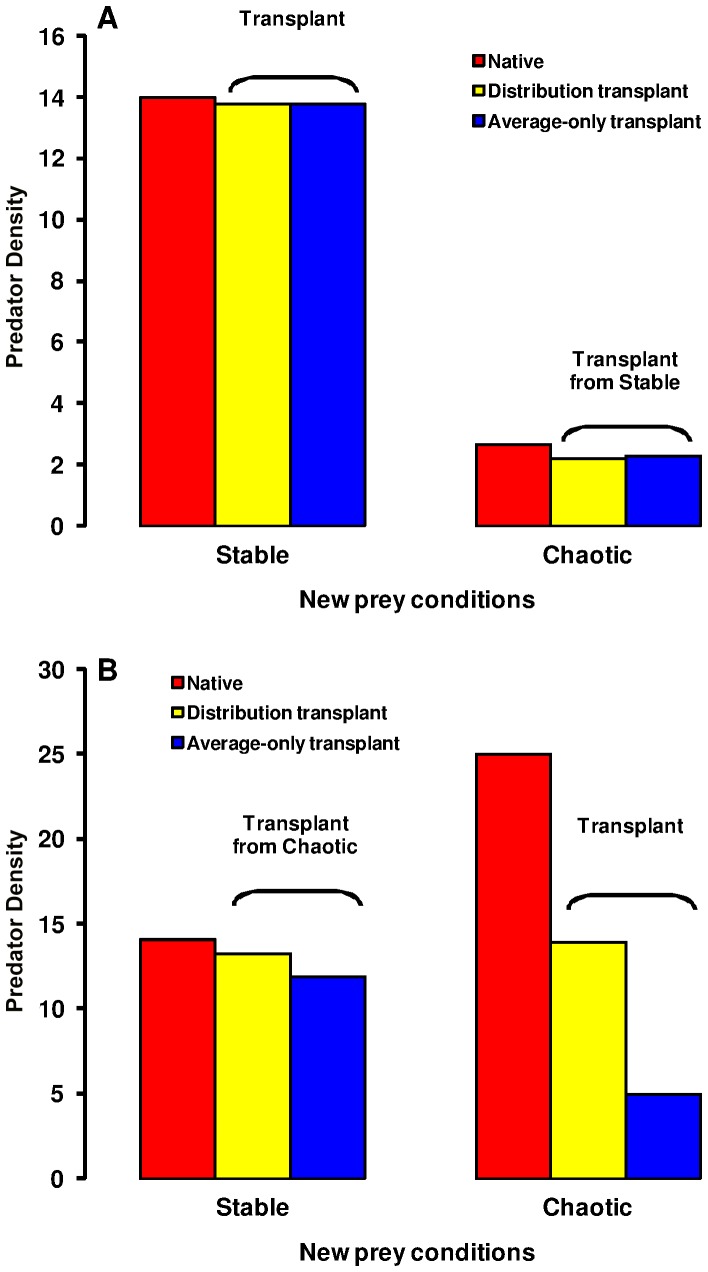
Variability in emigration probability and transplant success. Predator population size following transplant experiments between stable and chaotic prey landscapes involving predators with average-only emigration propensity or predators with their evolved distribution of emigration probabilities. In (A) the results are for the model where only emigration probability evolves. In (B) the results are for the more complex dispersal strategy where emigration rate evolves along with a stopping rule based on the ratio of prey to predators. These results are for the same parameter values as used in Fig. 5.

When a predator experienced an abrupt change in the underlying prey dynamics, the predator’s population size could be substantially compromised by a mismatch between the dispersal strategy which was evolved under one prey condition and that which would serve it best under the new conditions (Figs. 5 and 6). While predator transplanted population size was typically somewhat reduced compared to the native one, regardless of the prey conditions from which and to which individuals were moved, the most substantial negative impacts were experienced by populations moving from stable to unstable (either cyclic or chaotic) conditions. A population experiencing a transition from a chaotic prey environment to a stable environment could suffer, due to its maladapted (too high) dispersal strategy, roughly a 10% reduction in population size relative to a population which evolved under stable prey conditions, and this was true both when emigration probability alone evolved (Fig. 6A) and when the emigration probability and stopping rules evolved jointly (Fig. 6B). However, the reverse transition in prey dynamics, from stable to chaotic conditions, resulted in an even greater population decline (>70% for the joint evolution of emigration probability and stopping rule) relative to when it was well adapted to the unstable prey dynamics. Similar relative reductions resulted for a transition from stable to cyclic conditions. Note in Fig. 6 the substantially higher predator population densities obtained in complex prey landscapes by predator populations that used multiple steps (Fig 6B) than by those only moving to a nearest neighbour cell (Fig 6A). Active searching for prey resources resulted in much larger predator populations.

The extent of inter-individual variability in dispersal strategy (both emigration probability and stopping rules) at the time of transition to new prey conditions can have considerable influence on the predator’s ability to respond to the change. This is demonstrated by comparing the results of simulations within which we introduced 1000 individuals all adopting the population mean strategy with those where we introduced 1000 individuals randomly sampling inter-individual variability in dispersal strategies. In both cases, we disabled mutations at the point of introduction so that we were looking at the role of existing inter-individual variation in strategies rather than variation generated post introduction by novel mutations. The inter-individual variation present within a population that was transferred to new prey conditions provides some capacity for an improved ecological response through assortment of the variation in dispersal rules, When only emigration rate evolved, despite there being quite considerable variability (Fig. 1 inset), the difference in population size obtained in simulations with and without the inter-individual variability was quite small (Fig. 6A). However, the difference in population size obtained with and without inter-individual variability was much greater when the more complex dispersal strategy was applied (Fig. 6B). Here, the transplant from stable to chaotic prey dynamics that included inter-individual variability performed substantially better than that where every introduced individual was given the population mean emigration rate (resulting population size was four times greater with the variability present). When the transplant was in the opposite direction, from stable to chaotic, there was also a considerable benefit of having the initial inter-individual variability, although in neither case was it entirely sufficient to enable full adaptation to the novel conditions.

## Discussion

There is abundant theory related to the evolution of dispersal [43]–[45] and in recent years this theory has moved from a typically narrow focus on emigration rates to consideration also of the transfer and settlement phases of the whole dispersal process [11]. However, relatively little work has previously considered how dispersal behaviour should evolve in the context of trophic interactions and the existing work has almost entirely focussed on the emigration phase, most often considering either global dispersal or nearest neighbour dispersal [12], [14], [16]. Here, we have established some general predictions on how a specialist predator’s dispersal (both emigration probability and flexible stopping rules) should depend upon the spatio-temporal dynamics of its key prey species. The main general result is clear: when spatio-temporal prey dynamics are more complex, we should expect greater predator emigration rates and increasingly selective settlement behaviour which might result in predators dispersing over longer distances. Another important general question, which has received surprisingly little attention is how does having a maladapted dispersal strategy impact on a species’ population dynamics? Here, we have demonstrated that a rapid change in the spatio-temporal prey dynamics of the kind recently reported for multiple species with cyclic dynamics [33] is likely to render a population’s evolved dispersal strategy maladapted. We also showed this can potentially have substantial implications for the regional abundance of a population.

In our model, as we change the reproductive rate of the prey, we see a strong response in the evolved dispersal strategy of the predator (Figs. 1, 2, 3). Most strikingly, as *r* is increased above the bifurcation point at 2.0, we see a rapid increase in the evolved emigration probability, a substantial change in the reaction norm used for stopping and an emergent increase in the mean number of steps taken by an individual. These patterns in dispersal rules and emergent outcomes are driven by a shift in the prey dynamics from stable equilibrium to cycles that can become organised as travelling waves, hence becoming information-rich for a dispersing predator, and eventually (at high *r*) turning into spatio-temporal chaos. As the local prey dynamics have increasingly high-amplitude cycles, the selection for emigration probability and more selective settlement is clearly increased and, when the dynamics become chaotic, selection is even more intense, maybe reflecting an increased need for a predator to explore the state of its prey base. However, it should be noted here that under the deterministic chaos created by the Ricker model, very high prey populations are followed by a crash, under which circumstances high dispersal is strongly favoured; under different forms of chaotic dynamics, prey would potentially have some runs of very high years, in which case non-dipersing predators would have an advantage. This result mirrors those found in single-species models where, as dynamics move from being inherently stable to inherently unstable, greater emigration rates and dispersal distances evolve [46], [47]. In our model, as prey dynamics become temporally complex, there is also greater spatial complexity with the formation of travelling waves and eventually, spatial chaos, a pattern of covariation predicted by theory ([34]and supported by empirical evidence e.g. [48]) (Fig. S1). In a complex prey environment, greater selectivity over a destination patch is favoured as it enables a predator lineage to track the spatially changing resource (the prey), provided the costs of dispersal are sufficiently low. Note that the decline in emigration probability as *r* increases up until the threshold at *r* = 2.0 is due to the increase in size of the prey population (which in this region of parameter space always exhibits stable equilibrium dynamics). This is consistent with the large body of previous theory [49], demonstrating that, in a spatio-temporally homogenous environment, we should expect a decline in emigration as the carrying capacity increases.

Having a maladapted dispersal strategy can have considerable consequences for a predator population. Here we show that the transition from stable to chaotic or complex prey dynamics is more harmful than the other way round. In this case, the predators may have both insufficiently high emigration probability and exhibit insufficiently discriminatory settlement behaviour to track the moving ridges of high prey abundance effectively. The population-level consequences are less severe when the shift in prey dynamics is from complex to stable, and it seems likely that this result will hold quite generally. On that basis, we predict that having an inflexible evolved dispersal strategy that is higher than optimal under current conditions will have less severe population consequences than having a strategy that is lower than optimal. Higher emigration probabilities and greater selectivity over destination patches tend to evolve when there is substantial temporal variability in local conditions and when local extinction is relatively common. Under these conditions, a population with low dispersal ability will perform poorly and potentially be at risk of extinction. However, a population that has evolved high emigration probability and highly selective patch choice is less threatened by a shift to more stable prey landscapes. Under these environmental conditions, the only likely penalty of high dispersal will be the excessive mortality of dispersers, and we would therefore not anticipate populations maladapted in this direction to be as vulnerable to extinction.

While motivated by empirical evidence, the models we presented here were deliberately abstract and general, providing a starting point from which further studies can add increasing biological realism. As in almost all previous theory on evolution of dispersal, we have used a model representing an organism with discrete and non-overlapping generations. Some of the predator species, about which there is current concern due to changes in prey dynamics (e.g. arctic foxes *Alopex lagopus* and Tenglman owls *Aegolius funereus* preying on vole and lemmings in Fennoscandia [32], [50]), are clearly not well represented by such a model. There is a real and pressing need for the development of models, incorporating life-history evolution, that better represent longer-lived species with overlapping generations. With such a model, it would also be possible to ask under what spatio-temporal prey conditions we might expect a species to hold a territory and therefore to balance the benefits of skipping reproduction in low prey years against behaving nomadically and dispersing every year in an attempt to track the travelling wave of high resource availability [30].

In this contribution, we have assumed that the predator population exerts no influence on the prey dynamics. Clearly for many systems, this assumption may not hold true. There is thus considerable scope for future work that explores the evolution of dispersal strategies in fully coupled predator-prey systems. In particular, it will be interesting to explore the joint evolution of informed dispersal strategies of predators and their prey, asking how both species may use one another’s local densities as cues to inform emigration and settlement decisions.

In conclusion, we have demonstrated that the population dynamics of one species can have a major impact on the evolution of dispersal strategy in a second species through trophic interactions. This can have considerable implications for population vitality and viability. We suggest that the role of species interactions in driving dispersal is likely to be widespread and is a field deserving of greater theoretical and empirical attention.

## Supporting Information

Figure S1 **Spatial and temporal dimensions of prey landscapes.** (A) Time-series of prey density over 100 time-steps (from one randomly chosen cell in the lattice) for three different dynamics, respectively from left to right: Stable (*r* = 2.0, *m* = 0.05), Cyclic (*r* = 3.0, *m* = 0.1), Chaotic (*r* = 4.0, *m* = 0.4). (B) Maps of prey density taken from the original lattice (100 × 133 cells).
(TIF)

Figure S2 **The evolution of a reaction norm that determines patch selection results in spatially heterogeneous costs of dispersal across a spatio-temporally complex prey landscape.** The number of predators breeding in each cell at time T (B) is related to the prey density at time T (A), but by no means perfectly. However, being born in a good location does not necessarily imply that post-natal dispersal will be successful (C), as the local prey landscape may have changed dramatically by the time of dispersal (D). In particular, juvenile predators born under good conditions on the right-hand side of the region have relatively low dispersal success, because of the widespread ‘crash’ in prey density following a peak generation. A randomly-selected region of 20×20 cells is shown at T = 500 generations after predator establishment, where prey *r* = 3.5 and predator dispersal mortality *c_step_* = 0.05.
(TIF)
