# Supplementary figures and images for: Evolution of Predator Dispersal in Relation to Spatio-Temporal Prey Dynamics: How Not to Get Stuck in the Wrong Place!
Source: PLoS One. 2013 Feb 11;8(2):e54453. doi: 10.1371/journal.pone.0054453 (PMC3569443; doi:10.1371/journal.pone.0054453)

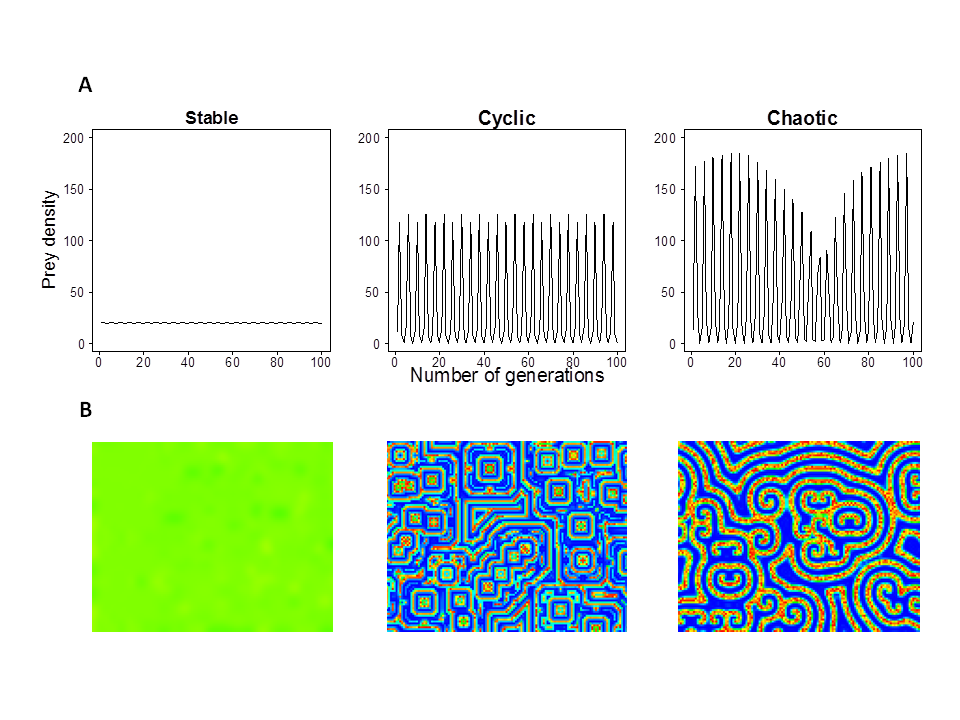

Supplement: Figure S1 — Spatial and temporal dimensions of prey landscapes. (A) Time-series of prey density over 100 time-steps (from one randomly chosen cell in the lattice) for three different dynamics, respectively from left to right: Stable (r = 2.0, m = 0.05), Cyclic (r = 3.0, m = 0.1), Chaotic (r = 4.0, m = 0.4). (B) Maps of prey density taken from the original lattice (100 × 133 cells). (TIF) [file pone.0054453.s001.tif]

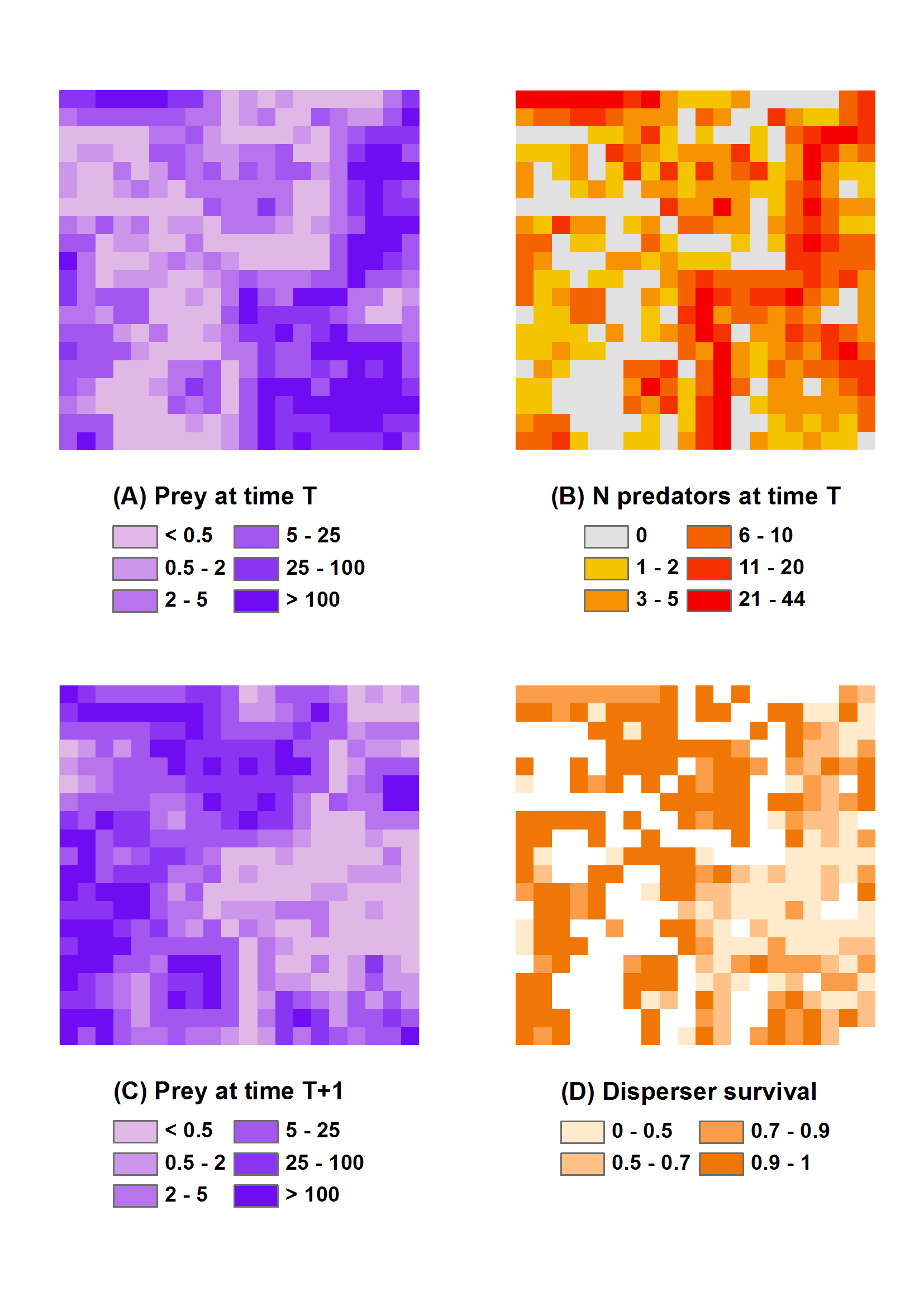

Supplement: Figure S2 — The evolution of a reaction norm that determines patch selection results in spatially heterogeneous costs of dispersal across a spatio-temporally complex prey landscape. The number of predators breeding in each cell at time T (B) is related to the prey density at time T (A), but by no means perfectly. However, being born in a good location does not necessarily imply that post-natal dispersal will be successful (C), as the local prey landscape may have changed dramatically by the time of dispersal (D). In particular, juvenile predators born under good conditions on the right-hand side of the region have relatively low dispersal success, because of the widespread ‘crash’ in prey density following a peak generation. A randomly-selected region of 20×20 cells is shown at T = 500 generations after predator establishment, where prey r = 3.5 and predator dispersal mortality cstep = 0.05. (TIF) [file pone.0054453.s002.tif]
